# Using Mobile Phones for Activity Recognition in Parkinson’s Patients

**DOI:** 10.3389/fneur.2012.00158

**Published:** 2012-11-07

**Authors:** Mark V. Albert, Santiago Toledo, Mark Shapiro, Konrad Kording

**Affiliations:** ^1^Sensory Motor Performance Program, Rehabilitation Institute of ChicagoChicago, IL, USA; ^2^Department of Physical Medicine and Rehabilitation, Northwestern UniversityChicago, IL, USA

**Keywords:** mobile phone, accelerometer, activity recognition, Parkinson’s disease

## Abstract

Mobile phones with built-in accelerometers promise a convenient, objective way to quantify everyday movements and classify those movements into activities. Using accelerometer data we estimate the following activities of 18 healthy subjects and eight patients with Parkinson’s disease: walking, standing, sitting, holding, or not wearing the phone. We use standard machine learning classifiers (support vector machines, regularized logistic regression) to automatically select, weigh, and combine a large set of standard features for time series analysis. Using cross validation across all samples we are able to correctly identify 96.1% of the activities of healthy subjects and 92.2% of the activities of Parkinson’s patients. However, when applying the classification parameters derived from the set of healthy subjects to Parkinson’s patients, the percent correct lowers to 60.3%, due to different characteristics of movement. For a fairer comparison across populations we also applied subject-wise cross validation, identifying healthy subject activities with 86.0% accuracy and 75.1% accuracy for patients. We discuss the key differences between these populations, and why algorithms designed for and trained with healthy subject data are not reliable for activity recognition in populations with motor disabilities.

## Introduction

Accurately tracking the activities of patients with motor disabilities has the potential to better inform patient care. With more precise, objective measures treatment alternatives can be evaluated more definitively. This is particularly important in motor disabilities such as Parkinson’s disease (PD) that respond to an increasing variety of treatment options, including drugs and various exercise therapies (Palmer et al., 1986; Schenkman et al., 1998; Goodwin et al., 2008; Dibble et al., 2009). Quantifying symptoms both in the clinic and at home has the potential to provide functional measures better associated with quality of life (Ellis et al., 2011).

Current means of evaluating patient mobility are limited. Clinical evaluations require a patient to travel to the location of a health care provider, and testing is expensive in terms of money and clinician time. This limits the frequency of clinical evaluations to only a few times per year for personal needs and only a few times per week during research studies. For better temporal resolution, studies often turn to patient journaling. Asking patients to periodically indicate their activities suffers from a number of problems. First, it is subjective, leading to changes based on the mental state of the subject. Also, because of the inconvenience to patients, it is difficult to achieve high compliance, with one study indicating only an 11% compliance rate using journaling (Stone et al., 2003). It would be helpful to develop a measure of evaluating patient mobility that is both frequent and convenient for the subject.

Dedicated accelerometers are an inexpensive, standardized way of measuring movement (Mathie et al., 2004b). These components can cost as little as one dollar, and can measure movement in any direction as well as orientation relative to gravity. For example, by attaching an accelerometer to a shoe, one can estimate the amount of time running and walking based on the periodic movements. For activity recognition, there have been many studies which place these accelerometers at specific locations on the body – including the head, chest, arm, foot, and thigh (reviewed in Kavanagh and Menz, 2008). The advantage of placing accelerometers at such locations is more consistent signals across individuals. Although such accelerometers are inexpensive, they are often dedicated equipment that has to remain attached at a particular location on the body to be effective. This is often an impractical inconvenience for long-term use.

Modern mobile phones have built-in accelerometers that can be used to track movements without the need for an additional device (Brezmes et al., 2009; Gyorbiro et al., 2009; Ryder et al., 2009; Fernandes et al., 2011). They have their own power sources, memory storage capabilities, and can transmit data wirelessly. Patients could simply download an app onto their smartphone enabling data collection and analysis. Mobile phones allow automatic, convenient, real-time monitoring, and recording, which can be invaluable to large-scale studies and personal patient health monitoring.

Unfortunately, applying current mobile phone strategies to populations with motor disabilities is challenging. For example, PD symptoms include tremor, slowed motion (bradykinesia), rigid muscles, loss of common automatic movements, and impaired posture (Jankovic, 2008). These symptoms all can adversely affect activity recognition. Activity recognition strategies have been tailored specifically for the elderly (Najafi et al., 2003), individuals with muscular dystrophy (Jeannet et al., 2011), and even PD (Salarian et al., 2007), but each of these studies were done with accelerometers at standardized locations or using multiple sensors throughout the body. Performing activity recognition for a population with a motor disability when carrying the phone naturally in pockets or belt clips provides additional challenges.

Here we show how modern machine learning techniques can quantify the movements of PD patients that carry mobile phones. We first collect data from both healthy subjects and PD patients performing a standard set of activities. From this data, we analyze the precision of activity recognition both within and across the two groups. Ultimately, we demonstrate an approach to activity recognition using mobile phone devices for patient populations, allowing us to better monitor patient responses to treatment in future studies.

## Materials and Methods

Eighteen healthy subjects and eight PD patients were recruited for this study. The eighteen healthy subjects, having had no previous history of a movement disorder, came from two groups, 13 younger subjects (6M/7F, 25.1 ± 3.0 years), and five older subjects (5F, 53.4 ± 7.4 years). The PD patients were mild and moderately affect patients in Hoehn and Yahr stage 1–3 (7F/1M, 67.0 ± 8.1 years, median ± range). Patients were recorded while taking their usual medications (ON-med condition). Many patients presented mild dyskinesias during the course of the experiment. Written, informed consent was obtained for all subjects. The Northwestern University Institutional Review Board approved this study.

All subjects were instructed to carry T-mobile G1 phones running Android OS version 1.6 in their front pockets. These phones have a standard built-in tri-axial accelerometer with a range of ±2.8 g. The sampling rate was variable between 15 and 25 Hz depending upon the amount of movement.

**Figure 1:**
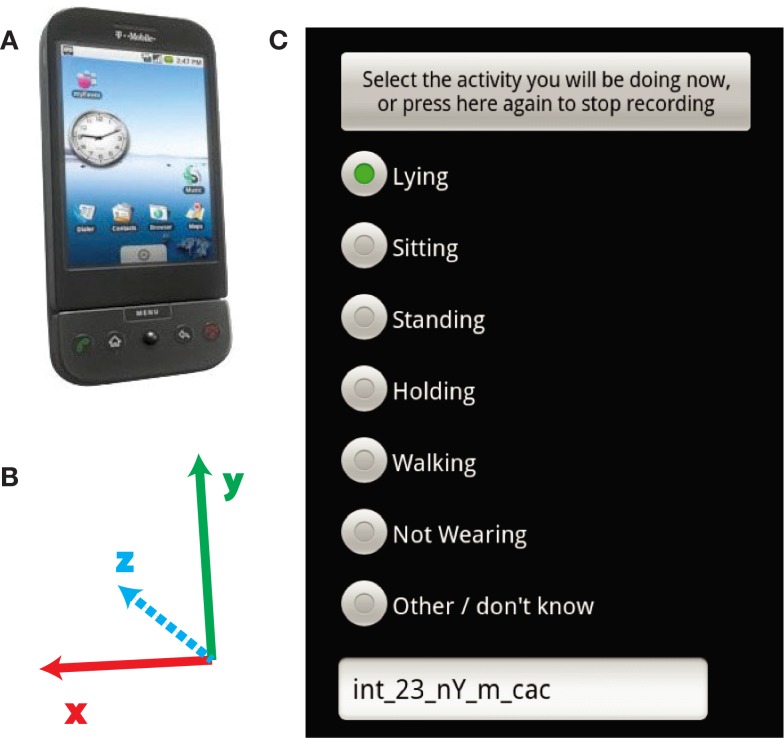
**Recording device and software**. **(A)** The subjects carried T-mobile G1 android phones in their pockets. **(B)** The axes of the accelerometer relative to the orientation of the phone in **(A)**. **(C)** The screen which subjects selected which activity they were performing.

Subjects were instructed to perform a number of different activities, each for at least 1 min. Before each activity, the subject would select the activity on a specially designed phone app (Figure 1) with the experimenter present to minimize errors. The accelerations were labeled according to the activity they were performing. The activities were performed in a laboratory setting in the order shown below. For a few initial subjects the activities began and ended with an additional lying down activity, but the protocol was simplified by removing this activity for all subjects later on. We also repeated activities to get more recordings with the phone in slightly different positions on the subjects.

1. sitting
2. standing
3. holding the phone (standing with arms bent forward)
4. walking
5. not wearing (set on a table)
6. walking
7. holding the phone
8. standing
9. sitting

### Data processing and classification

The accelerometer signals were preprocessed using the following procedure: the recordings were segmented into 10 s clips performing a given activity for the entire duration. The first and last samples were removed from the ends of the recording to allow time for the phones to enter and be removed from the pockets of subjects. Three thousand three hundred and eighty eight samples were recorded from healthy subjects, while 2184 total samples were recorded from PD patients. The subject and researcher both observed labeling during recording, ensuring validity of training, and test data. Misclassified samples were only checked for artifacts. The phone accelerometer values were linearly interpolated from a variable rate between 15 and 25 Hz to match 20 Hz.

The subjects naturally placed the phone in their pockets in the following possible orientations, due to the elongated rectangular shape of the phones.

1. screen in/right side up
2. screen in/upside down
3. screen out/right side up
4. screen out/upside down

In the accelerometer readings, each of these orientations only differs by the signs of the axes. For example, flipping the phone right side up to upside down (e.g., orientation #1–#2) changes the sign of the *x* and *y* axes, while turning the screen inward (e.g., #1–#3) changes the sign of axes *x* and *z* axes. To correct for these different phone orientations, we generated three additional samples for each recorded sample by a coordinate transform that effectively flips the phone 180° along each of its three axes.

From these 10 s clips features were extracted, as summarized in Table 1.

**Table 1:** **Features used for activity recognition**.

Description	Total number of values
Mean, absolute value of the mean	6
Moments: standard deviation, skew, kurtosis	9
For the change in acceleration: mean, standard deviation, skew, kurtosis	12
Root mean square	3
Smoothed root mean square (5 pt kernel, 10 pt kernel)	6
Extremes: min, max, abs min, abs max	12
Histogram: includes counts for −4 to 4 *z*-score bins	27
Fourier components: 32 samples for each axis	96
Overall mean acceleration	1
Cross product means: *xy*, *xz*, *yz*	3
Abs mean of the cross products	3

Two popular algorithms were used for classification: support vector machines (SVM; Chang and Lin, 2011) and sparse (regularized) multinomial logistic regression sparse multinomial logistic regression (SMLR; Krishnapuram et al., 2005). Both techniques have been successfully applied in a large number of machine learning classification problems with a great deal of practical success on large feature sets. The hyperparameters for both classifiers were found by a grid search of 10× where *x* is an integer between −5 and 5 and selecting the maximum cross-validated error in predicting the healthy subject labeled activities. Given the size of the data set used for cross validation this procedure was not expected not lead to noticeable over-fitting for the hyperparameters. For SMLR, the coefficient for the regularization term during optimization, λ, was 0.0001. For SVM, we normalized each feature to have 0 mean and unit variance. We applied radial basis functions, giving us two hyperparameters – the soft slack variable, *C*, and the size of the Gaussian kernel, γ. The values found by cross validation were *C* = 1 and γ = 0.1 for the across subjects validation and *C* = 10 and γ = 1 for the 10-fold validation.

## Results

To examine our ability to classify activities in a patient population, we collected data on both PD patients and healthy subjects, as detailed in the Section “Materials and Methods.” Subjects were instructed to carry mobile phones in their front pants pocket while performing a series of activities (Figure 1C). We applied two different classifiers, SVM and SMLR, to classify the activities. Our intention is to demonstrate the importance of using classifiers trained with data specifically from patient populations.

**Figure 2:**
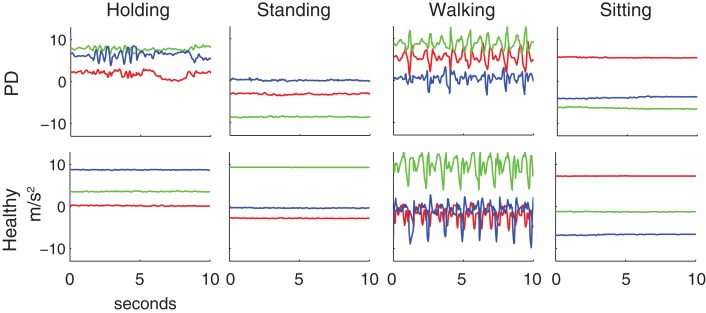
**Typical examples of accelerometer readings for Parkinson’s patients and healthy subjects for the four activities studied**. Red, green, and blue lines are the *x*, *y*, and *z*-axis accelerations, as specified in Figure 1B. The patient shown here exhibited dyskinesia in the arm that is clearly visible while holding the phone and somewhat visible during standing and sitting. The patient also had an irregular gait cycle during walking.

The activities subjects performed have characteristic patterns in the accelerometer signals. Recordings were made from the three-axis accelerometers in the phones. The orientation of the phone determined the orientation of the accelerometer axes (Figure 1B). There are also visible differences in the data of PD patients compared to healthy subjects. Example clips from these activities (Figure 2) show the presence of dyskinesias in one PD patient. It can also be observed in the accelerations that walking is often less periodic for PD patients than healthy subjects. Such differences can lead to errors in classification if features such as periodicity or vibrations are used for prediction. Although the movements are related between groups, the exact characteristics of those movements have enough differences between the populations to examine the effect of these differences on classification accuracy.

Unlike many studies which classify signals based on only a few, specific features, we did not seek individual features that could be used for classification. For example, knowing if someone is standing, sitting, or holding the phone can depend not only on the orientation of the phone, but also on the amount of vibration in the movement. Instead of searching for particular features with clear, independent differences between activities, we chose to apply the standard, state-of-the-art machine learning approach: we constructed a large feature set and had the algorithms select how to combine and weigh the features appropriately.

First, we wanted to compute a classification accuracy measure that can be compared across studies. To do this, we used 10-fold cross validation, selecting every 10th sample for the test set. This accuracy is expected to be fairly high considering movement patterns specific to individual subjects were in both the training and test sets. For SVM classification, this lead to a 96.1% accuracy for healthy subjects (Table 2), and a 92.2% accuracy for PD patients (Table 3). Similar results were found for SMLR (89.7% for healthy, and 84.7% for PD patients) so only the SVM results are shown for clarity.

**Table 2:** **Classification matrix for healthy subjects with 10-fold cross validation**.

Activity	Walking	Standing	Holding	Sitting	Not wearing
Walking	**908**	0	0	0	0
Standing	0	**708**	4	8	0
Holding	2	22	**768**	0	4
Sitting	4	32	48	**652**	0
Not wearing	0	0	4	4	**220**

*96.1% overall accuracy for 18 healthy subjects*.

**Table 3:** **Classification matrix for PD patients with 10-fold cross validation**.

Activity	Walking	Standing	Holding	Sitting	Not wearing
Walking	**662**	0	6	10	34
Standing	0	**400**	0	16	0
Holding	4	0	**376**	32	4
Sitting	10	24	2	**396**	4
Not wearing	8	0	12	4	**180**

*92.2% overall accuracy for eight PD subjects*.

We sought to quantify the effect of differences between movements made by Parkinson’s patients and healthy subject on the classification algorithm. Unlike previous studies that have analyzed the difference from healthy subjects for particular features (Salarian et al., 2007; Jeannet et al., 2011), we directly trained the classifiers using healthy subject data, and applied the classifiers to patient recordings to observe the effect of those differences. From this, we achieved a much lower accuracy of 60.3% using SVM’s (Table 4) and 63.5% using SMLR. The lower accuracy is indicative of the difference between the populations and the need to find another, more accurate way to identify patient activities.

**Table 4:** **Classification matrix for PD patients using healthy subject training data**.

Activity	Walking	Standing	Holding	Sitting	Not wearing
Walking	**428**	96	44	120	24
Standing	0	**308**	36	72	0
Holding	36	0	**180**	184	16
Sitting	0	56	104	**276**	0
Not wearing	0	0	48	32	**124**

*60.3% overall accuracy for 18 healthy subjects for training and eight PD patients for testing*.

One entry in the table is made for every 10 s sample. The rows represent the activity being performed and the columns represent the prediction according to the algorithm. Overall accuracy is the sum of the correct classification (diagonal in bold) compared to the sum of all entries in the entire table.

We sought to better understand the large difference in accuracy for the within vs. across population results. Ten-fold or leave-one-out cross validation techniques do not remove the effect of the same individual that may have significantly distinct movement pattern from others. Using 10-fold cross validation would still allow such idiosyncratic movements of individuals to be part of the training and test sets. We wanted a measure that would indicate the accuracy of the algorithm if it were applied to subjects after training. For this we performed subject-wise cross validation for the 18 healthy subjects and found an accuracy of 86.0% for SVM’s and 85.2% for SMLR. Note that because much of the variation in movements is across subjects, this accuracy is much lower than that of the 10-fold cross validation. Table 5 presents a breakdown of the classification for SVM’s, with SMLR appearing similar.

**Table 5:** **Classification matrix for healthy subjects with subject-wise cross validation**.

Activity	Walking	Standing	Holding	Sitting	Not wearing
Walking	**900**	4	0	0	4
Standing	8	**676**	0	36	0
Holding	0	88	**624**	40	44
Sitting	4	120	120	**492**	0
Not wearing	0	0	8	0	**220**

*86.0% overall accuracy for 18 healthy subjects*.

To consider the ability of this approach to be adapted to PD patients by using patient data, we also analyzed the PD patients separately. Similar to the healthy subjects, we applied subject-wise cross validation on the PD patient data alone. Using SVM’s, the accuracy was 75.1% (Table 6) and using SMLR it was 76.0%. Although this is lower than the previous percentage for healthy subjects, this is expected as PD patients movements vary more significantly across subjects. Most importantly, when considering predictions across subjects, training using patient data led to a significantly better prediction than training using healthy data alone.

**Table 6:** **Classification matrix for PD patients with subject-wise cross validation**.

Activity	Walking	Standing	Holding	Sitting	Not wearing
Walking	**588**	8	80	4	32
Standing	8	**400**	4	4	0
Holding	20	4	**296**	56	40
Sitting	8	84	132	**208**	4
Not wearing	0	0	56	0	**148**

*75.1% overall accuracy for eight PD subjects*.

## Discussion

We applied machine learning to signals from mobile phones to classify the activities of people with PD. Instead of handpicking the most relevant features and comparing them, we used a large feature set and had the relevant features selected by the machine learning algorithms. Because many algorithms depend on the training data, these methods were not expected to test well for populations with unique movement patterns. This was done using mobile phones carried naturally in pants pockets. Though this natural way of carrying was expected to lower accuracy values, it is more indicative of expected accuracy if this research is to be applied to studies with patient groups.

The most accurate methods of data collection for activity recognition rely on multiple sensors. Often this involves accelerometers, as they are small, relatively inexpensive, and register both movement and orientation to gravity. Some systems have integrated temperature, compass, light, and sound sensors on the waist (Choudhury et al., 2008) or a similar collection of multimodal sensors on the wrist (Maurer et al., 2006; Gyorbiro et al., 2009). For accelerometer-only arrays, multiple sensors may be placed throughout the body – anywhere from three to five locations (Bao and Intille, 2004; Tapia et al., 2007; Krishnan and Panchanathan, 2008) or more. Mannini and Sabatini (2010) provide a review of these approaches.

There are simpler alternatives to using multiple sensors, improving the convenience, cost, and compliance rates. The most common approach is to use a single, waist-mounted accelerometer. This approach has been analyzed on very specific sets of instructed activities with over 98% accuracy (Mathie et al., 2004a; Mathie et al., 2004b; Ravi et al., 2005; Lee et al., 2009). High accuracy ratings were possible in part due to the fixed location of the accelerometers on the body, the use of within-subject vs. across-subject cross validation, and the artificial nature of instructed movements. Signals from walking, sitting, and standing are necessarily more repeatable when in a consistent lab setting following instruction. For comparison, when subjects simply wore such a device for 24 h, with more natural activities, accuracy was closer to 80% (Long et al., 2009). Single, waist-worn accelerometers have been well-studied in the domain of activity recognition, but may need consistent placement for high accuracy.

Unlike dedicated accelerometers, some people already consistently carry mobile phones, making them a convenient platform for recording movements. Most smartphones have built-in accelerometers and are often worn on the person, similar in principle to previous work on accelerometry. Mobile phones have built-in communication protocols that allow simple data logging to the device and wireless transmission. This permits real-time response, or in an experimental setting, compliance verification. Because mobile phones are widely adopted, compliance without verification is already high, as people are used to carrying them. Due to these advantages, mobile phones have the promise to provide a convenient, inexpensive, and objective means to detect the activities of people.

Mobile phones have been used to classify activities of healthy subjects (Bieber et al., 2009; Brezmes et al., 2009; Gyorbiro et al., 2009; Ryder et al., 2009; Wang et al., 2009; Yang, 2009; Kwapisz et al., 2011). Common activities include walking, jogging/running, standing, sitting, and using stairs. The choice of activities influences accuracy rates, and also because most rates in these previous studies are not subject-wise cross-validated, applicability across subjects is more difficult to interpret. In Kwapisz et al. (2011), healthy subjects were instructed to carry the phone in their left pocket and perform a specific set of activities; all activities except stair climbing were classified with at least 90% accuracy. Other studies found similarly high accuracy but with different classification techniques (Brezmes et al., 2009; Ryder et al., 2009; Yang, 2009). In Wang et al. (2009), classes were divided as still, walking, running, or in a vehicle, which simplified classification which was done using microphones and GPS as well as accelerometer readings. In Yang (2009), a preprocessing technique was used which converted the axes from phone-specific to phone-independent coordinates based on orientation of gravity, providing 88–90% accuracy. While our results on healthy subjects are in line with previous studies, the central contribution of our paper is the careful analysis of precision of activity recognition in the context of PD.

### Patient populations

We chose to analyze the PD population for various reasons. Millions of people throughout the world are suffering from diseases that affect mobility. Many diseases, such as stroke, heart disease, or depression affect large populations but have a wide variety of causes, types, and symptoms. PD, on the other hand, is characterized by a number of common characteristics, which makes analysis easier across subjects (Gelb et al., 1999). Common symptoms such as tremor are visible in movements and lend themselves well to analyses using accelerometers in mobile phones (Joundi et al., 2011; Surangsrirat and Thanawattano, 2012). The PD population is also an important subgroup to consider as it also effects a relatively large population – approximately four million people globally (Dorsey et al., 2007).

There is another study that automatically classified and characterized postures and activities for a population of PD patients (Salarian et al., 2007). However, their results used within-subject cross validation and thus cannot speak to the across-subject generalization issue we are discussing here. Moreover, they used a set of accelerometers and gyroscopes instead of mobile phones. Our paper demonstrates the ability to use mobile phone recordings of acceleration to enable quality activity recognition with PD patients.

There are a few limitations to the interpretation of our results to address. For our healthy subjects, we used a population of both younger and older subjects, instead of age-matched controls. Some of the difference between the groups can be age-related, however we believe this effect was minor compared to the effect of PD on patient movements. Also, the PD group was relatively small (eight subjects) and heterogeneous (Hoehn and Yahr stage 1–3), however even from this heterogeneous group we note a significant improvement by using PD training data. Lastly, because we had both the researcher and subject observing, we relied on the recording procedure for accurate activity labeling. Subjects did not always perform the instructed actions in a typical fashion (e.g., moving feet while standing, stopping briefly while walking, etc). Instead of removing possible inconsistencies by hand, and thus affecting the validity of this approach, we retained all samples in the data set. Despite these limitations, the main conclusions of this study are supported.

There were two main goals for this study. First, we demonstrate how machine learning can be used to infer the activities of PD populations; the focus is not on particular, hand-picked features of movement, but on automated methods of weighing and combining those features. The second major goal was to highlight and quantify the effect of applying classifiers designed for healthy subjects on a PD patient population. A demonstrable drop in classification accuracy from 92.2 to 60.3% makes this point clear; it is important to use tools and analyses designed for specific patient populations. Although this study is not thorough enough to validate this classification method for clinical practice, it does demonstrate a strong benefit of machine learning, and a caution for clinicians who may want to use any activity recognition methods designed for healthy subjects.

The ultimate objective of therapies is to improve patient quality of life and activity tracking is an additional way of quantifying this. Such quantitative evaluation techniques could help clinicians test and optimize aspects of many therapies for motor disorders. By only downloading an application, mobile phones can record a person’s movements, greatly simplifying the study design and improving compliance. This information can be of personal or community medical use, improving evaluation of patient outcomes in therapeutic interventions. It is clear that populations with motor impairments require special consideration in approaches that analyze movement patterns. Mobile phones provide a means of tracking movements in an objective, convenient, and inexpensive way. The extent to which leveraging these qualities can improve and enable new therapeutic approaches is an area of further research.

## Conflict of Interest Statement

The authors declare that the research was conducted in the absence of any commercial or financial relationships that could be construed as a potential conflict of interest.
